# Osbpl8 Deficiency in Mouse Causes an Elevation of High-Density Lipoproteins and Gender-Specific Alterations of Lipid Metabolism

**DOI:** 10.1371/journal.pone.0058856

**Published:** 2013-03-15

**Authors:** Olivier Béaslas, Jari Metso, Eija Nissilä, Pirkka-Pekka Laurila, Essi Kaiharju, Krishna Chaithanya Batchu, Leena Kaipiainen, Mikko I. Mäyränpää, Daoguang Yan, Helena Gylling, Matti Jauhiainen, Vesa M. Olkkonen

**Affiliations:** 1 Minerva Foundation Institute for Medical Research, Helsinki, Finland; 2 Department of Chronic Disease Prevention, Public Health Genomics Research Unit, National Institute for Health and Welfare, and Institute for Molecular Medicine Finland (FIMM), Helsinki, Finland; 3 Institute of Biomedicine, Anatomy, University of Helsinki, Helsinki, Finland; 4 Institute of Biomedicine, Department of Biochemistry and Developmental Biology, University of Helsinki, Helsinki, Finland; 5 Division of Internal Medicine, Department of Medicine, University of Helsinki, Helsinki, Finland; 6 Department of Pathology, Haartman Institute, University of Helsinki and HUSLAB Division of Pathology, Meilahti Laboratories of Pathology, Helsinki University Central Hospital, Helsinki, Finland; 7 Department of Biology, Jinan University, Guangzhou, China; Harvard Medical School, United States of America

## Abstract

OSBP-related protein 8 (ORP8) encoded by Osbpl8 is an endoplasmic reticulum sterol sensor implicated in cellular lipid metabolism. We generated an Osbpl8^−/−^ (KO) C57Bl/6 mouse strain. Wild-type and Osbpl8KO animals at the age of 13-weeks were fed for 5 weeks either chow or high-fat diet, and their plasma lipids/lipoproteins and hepatic lipids were analyzed. The chow-fed Osbpl8KO male mice showed a marked elevation of high-density lipoprotein (HDL) cholesterol (+79%) and phospholipids (+35%), while only minor increase of apolipoprotein A-I (apoA-I) was detected. In chow-fed female KO mice a less prominent increase of HDL cholesterol (+27%) was observed, while on western diet the HDL increment was prominent in both genders. The HDL increase was accompanied by an elevated level of HDL-associated apolipoprotein E in male, but not female KO animals. No differences between genotypes were observed in lecithin:cholesterol acyltransferase (LCAT) or hepatic lipase (HL) activity, or in the fractional catabolic rate of fluorescently labeled mouse HDL injected in chow-diet fed animals. The Osbpl8KO mice of both genders displayed reduced phospholipid transfer protein (PLTP) activity, but only on chow diet. These findings are consistent with a model in which Osbpl8 deficiency results in altered biosynthesis of HDL. Consistent with this hypothesis, ORP8 depleted mouse hepatocytes secreted an increased amount of nascent HDL into the culture medium. In addition to the HDL phenotype, distinct gender-specific alterations in lipid metabolism were detected: Female KO animals on chow diet showed reduced lipoprotein lipase (LPL) activity and increased plasma triglycerides, while the male KO mice displayed elevated plasma cholesterol biosynthetic markers cholestenol, desmosterol, and lathosterol. Moreover, modest gender-specific alterations in the hepatic expression of lipid homeostatic genes were observed. In conclusion, we report the first viable OsbplKO mouse model, demonstrating a HDL elevating effect of Osbpl8 knock-out and additional gender- and/or diet-dependent impacts on lipid metabolism.

## Introduction

Oxysterol binding protein (OSBP) is a cytoplasmic protein with affinity for a number of oxysterols [1], [2]. It localizes in a sterol-specific manner on Golgi membranes and regulates the trafficking of ceramide from the endoplasmic reticulum (ER) to the Golgi apparatus for sphinghomyelin synthesis [3]. OSBP also acts as a sterol-dependent scaffold that modulates the activity of extracellular signal-regulated kinases, ERK [4]. Families of proteins homologous to OSBP are present in most eukaryotic organisms. In humans and mice the gene/protein family consists of 12 members [5]–[7]. The mammalian OSBP-related proteins (ORPs) have been implicated as sterol sensors that regulate cellular functions ranging from sterol, sphingolipid and neutral lipid metabolism to vesicle transport and cell signaling [2], [8], [9].

ORP8 encoded by the Osbpl8 gene is a member of the ORP family, with a trans-membrane segment at its C-terminus specifying localization at the ER. We have previously reported that ORP8 affects in human THP-1 macrophages the expression of ATP-binding cassette transporter A1 (ABCA1) and cellular cholesterol efflux [10], and ORP8 knock-down in Raw264.7 macrophage leads to several alterations in the cellular lipidome, including increased levels of both free cholesterol and cholesterol esters [11]. Moreover, we characterized the function of ORP8 in hepatic cells and its interaction with the nucleoporin Nup62, and demonstrated that adenovirus-mediated ORP8 overexpression in mouse liver affects lipid metabolism *in vivo*, resulting in a decrease of plasma and liver tissue cholesterol and triglycerides (TG), putatively via a reduction of active nuclear sterol regulatory element binding proteins (SREBPs) [12]. We envision that ORP8 may, via Nup62 interaction, fine-tune the transcriptional control of cellular lipid metabolism. Another aspect of its function most likely involves targeting of the plasma membrane via the N-terminal pleckstrin homology domain region (T.Vihervaara and V.M.Olkkonen, unpublished), where ORP8 could play a role in non-vesicular communication between the ER and the plasma membrane with impacts on membrane lipid composition/lateral organization and signaling processes. Interestingly, Osbpl8 was recently found to be target of miR-143, a micro-RNA induced in the liver in genetic and dietary mouse models of obesity, and the hepatic ORP8 protein was shown to be down-regulated under these conditions [13]. Prompted by the findings suggesting a function of Osbpl8 in lipid homeostatic control, we characterize in the present study the impacts of Osbpl8 deficiency on mouse lipid metabolism. The mouse strain generated represents the first viable mammalian Osbpl gene knock-out model.

## Results

### Osbpl8^−/−^ (KO) Mice

**Figure 1:**
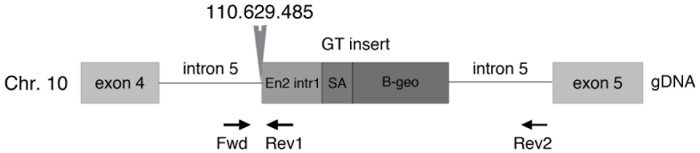
Gene trap (GT) insertion site in Osbpl8. Insertion of the pGT vector in the 5^th^ intron of the Osbpl8 gene in chromosome 10 generates a β-geo fusion mRNA transcript through the use of the En-2 splice acceptor (SA) contained in the vector. The insertion site (nt position indicated) was identified by PCR, using primers targeting gDNA in 5^th^ intron of Osbpl8 gene (Fwd), and in the 1^st^ intron of En2 (Rev1).

**Figure 2:**
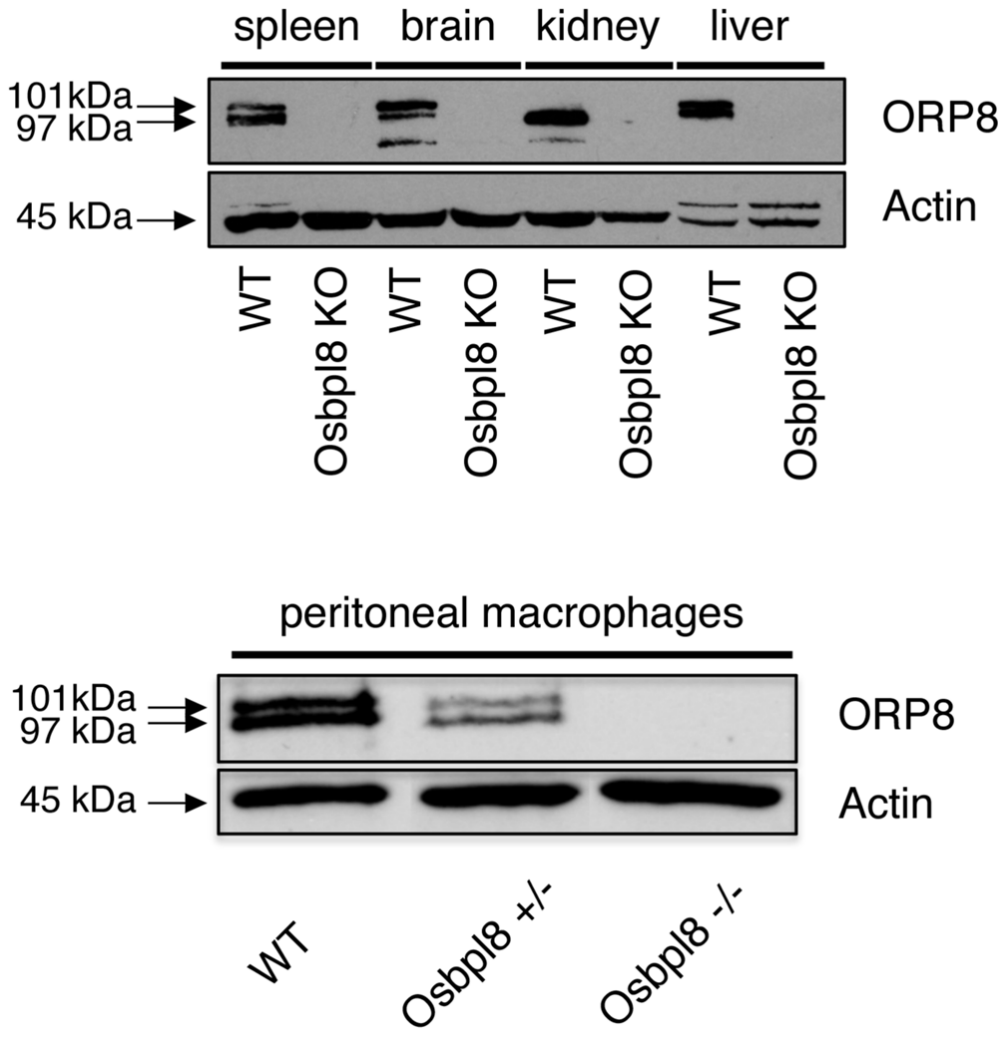
Western blot analysis of ORP8 in KO mouse tissues and peritoneal macrophage. Total protein specimens (20 µg protein/lane) of tissues with abundant ORP8 expression: spleen, brain, kidney, and liver (WT and Osbpl8KO animals), and of peritoneal macrophage (WT, Osbpl8+/−, and Osbpl8−/−) were analyzed by Western blotting with ORP8 and β-actin antibodies. ORP8 runs for an unknown reason as a doublet of bands with the apparent molecular masses of 97 and 101 kDa, as described in [10].

To gain insight into the function of Osbpl8/ORP8 *in vivo*, we generated Osbpl8 deficient mice by using gene trapped ES cells, in which a gene trap is inserted in exon 5 of the Osbpl8 gene (Fig. 1). The gene trapped Osbpl8 allele was brought into >98% C57Bl/6 genetic background. Successful knock-out was verified by Western analysis of tissues and peritoneal macrophages of wild-type (WT), Osbpl8^−/−^ (Osbpl8KO), or Osbpl8^+/−^ animals, by using antibodies specific for ORP8. The analysis revealed the absence of immunoreactive ORP8 protein in Osbpl8KO mouse tissues which in WT animals express the protein abundantly: liver, brain, kidney, spleen, and macrophages [10](Fig. 2). The macrophages of heterozygotic Osbpl8^+/−^ mice displayed an intermediate level of ORP8 protein as compared to the WT and KO animals. No apparent defects in general health or fertility were observed in the KO mice.

### Plasma Lipids/lipoproteins of the Osbpl8KO Mice

To investigate whether Osbpl8 deficiency causes changes in plasma lipids and lipoproteins, groups of 13-week old WT or Osbpl8KO mice were fed either regular chow or high-fat diet (21.2%% fat, 0.2% cholesterol) for 5 weeks. Fasting blood samples were withdrawn at the beginning (0 weeks) and the end of the 5-week dietary period, and plasma lipids (total cholesterol, TC; free cholesterol, FC; triglycerides, TG; choline-containing phospholipids, PL) were analyzed by enzymatic assays (Table 1). Significant increases of plasma TC, FC and PL were detected at the starting time point in both genders of the Osbpl8KO mice (age 13 weeks); Additionally, the TG were significantly increased in female KO mice relative to WT littermates. After further 5 weeks on chow diet (age 18 weeks) the male KO mice displayed elevated TC and FC, and a statistically non-significant tendency of PL increase as compared to WT littermates, while the females showed tendencies of TC, FC and PL elevation and a significant elevation of TG. After 5 weeks on Western diet both genders of KO mice displayed significantly elevated levels of plasma TC, FC, and PL as compared to WT littermates, while the TG were increased significantly in KO males. Quantification of apolipoprotein A-I (apoA-I) revealed in both genders a modest (16% for females; 10% for males) but statistically significant (p<0.05) elevation in Osbpl8KO mouse plasma at the starting point (Table 1). After the 5-week diet period, no consistent apoA-I elevation was observed in the Osbpl8KO mice, the only significant difference found being an elevated level in chow diet-fed KO females.

**Table 1:** Fasting plasma lipid concentrations of WT and Osbpl8KO mice before (n = 12) and after (n = 6) the diets.

t = 0	TC#	p##	FC	p	TG	p	PL	p	apoA-I§	p
FemaleWT	1.806±0.130		0.509±0.044		0.602±0.051		1.779±0.111		0.94±0.03	
Female KO	2.575±0.162	0.0013**	0.768±0.067	0.0043**	0.888±0.055	0.001**	2.3740±101	6.45E–4***	1.1±0.03	0.0024**
Male WT	2.309±0.096		0.769±0.030		1.074±0.071		2.555±0.110		1.27±0.03	
Male KO	3.442±0.104	5.75E–8***	1.181±0.044	3.10E–7***	1.076±0.0.47	0.983	3.262±0.081	4.58E–5***	1.39±0.04	0.026*
t = 5 wkChow diet										
Female WT	2.056±0.222		0.558±0.040		0.716±0.054		1.974±0.194		1.05±0.08	
Female KO	2.478±0.122	0.136	0.637±0.029	0.142	0.989±0.057	0.00605**	2.215±0.097	0.303	1.42±0.11	0.023*
Male WT	2.699±0.067		0.832±0.024		1.270±0.083		2.933±0.095		1.72±0.1	
Male KO	3.247±0.400	0.0119**	1.017±0.109	0.00391**	1.219±0.294	0.705	3.551±0.303	0.0999	1.45±0.16	0.17
t = 5 wkWestern diet										
Female WT	2.490±0.159		0.680±0.026		0.807±0.049		1.852±0.143		1.45±0.02	
Female KO	3.887±0.242	0.00106**	0.979±0.044	3.87E–4***	0.940±0.048	0.0824	2.491±0.200	0.0286*	1.51±0.07	0.47
Male WT	4.791±0.279		1.267±0.049		1.099±0.084		3.563±0.178		2.07±0.08	
Male KO	5.907±0.304	0.0224*	1.570±0.037	8.44E–4***	1.372±0.063	0.0283*	4.131±0.060	0.023*	2.22±0.17	0.43

# TC, total cholesterol; FC, free cholesterol; TG, triglycerides; PL, choline-containing phospholipids; mmol/L.

## T-test, comparison between genotypes; *p<0.05, **p<0.01, ***p<0.001.

§ apolipoprotein A-I; mg/ml.

**Figure 3:**
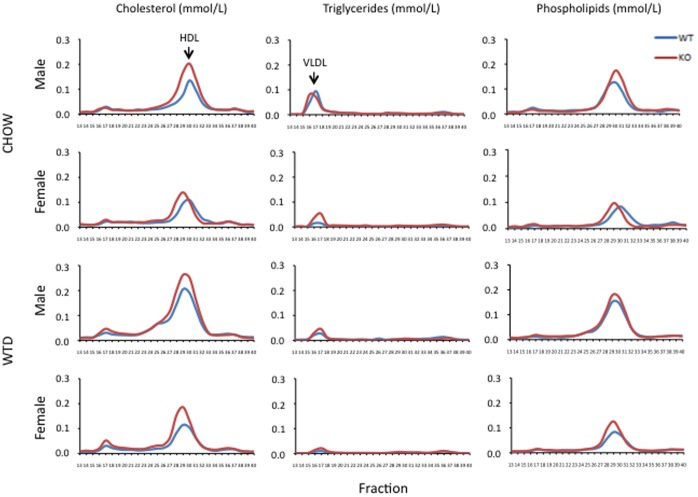
Effects of Osbpl8KO on the lipoprotein profile. Pools of mouse plasma were fractionated by size exclusion chromatography as specified in Material and Methods, followed by analysis of total cholesterol, triglycerides, and choline-containing phospholipids (identified at the top) in the fractions. The 5-week diet, chow (CHOW), or Western diet (WTD), as well as the gender, are indicated on the left. WT, blue; KO, red. Each panel represents an average of 2 pools analyzed, each consisting of 3 animals. Elution positions of HDL and VLDL are indicated in the first two panels.

**Figure 4:**
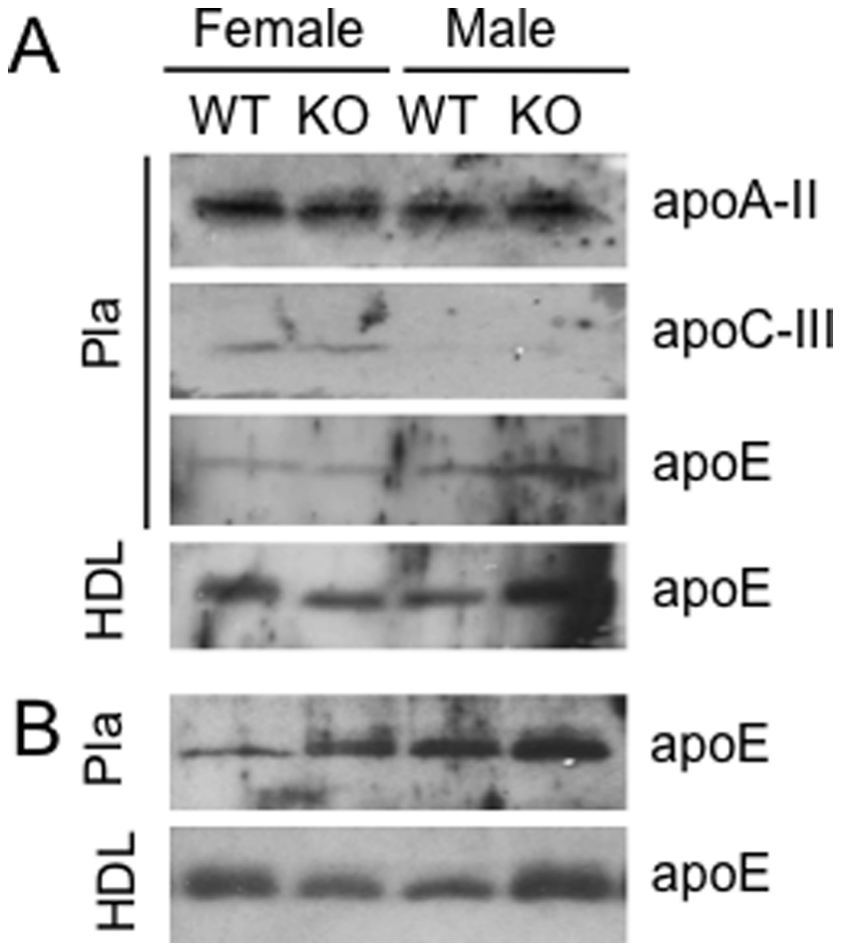
Western blot analysis of HDL apolipoproteins in WT and KO mouse plasma and high-density lipoprotein fractions. A. Chow diet-fed animals; ApoA-II, ApoC-III, and ApoE (identified on the right) in the fasting plasma (Pla) of WT and KO mice of both genders (identified at the top); Bottom panel, ApoE in the HDL peak fractions of plasma fractioned on a Suprose HR 6 10/30 column. B. Western diet-fed animals; ApoE in the plasma (Pla) and HDL fractions (HDL) of WT and KO mice of both genders (identified at the top). The plasma loading was 3 µl/lane of 1/40 dilution, and that of Superose 6 HR 10/30 HDL fractions 150 µl/lane concentrated by acetone precipitation. The analysis was carried out for pooled plasma and HDL fractions isolated from the same pools; Chow diet, 3 animals/pool; Western diet, 6 animals/pool.

Lipoprotein profile analysis was carried out to investigate in which lipoprotein class(es) the observed TC, PL and TG increases are localized. A marked increase of both TC and PL in the high-density lipoprotein (HDL) of Osbpl8KO male plasma was revealed as compared with WT controls after both chow (TC, +79%; PL, +35%; Fig. 3C,D) and Western diet (TC, +37%; PL, +21%; Fig. 3) feeding, and in female KO mice after the Western diet (TC, +58%; PL, +46%). After 5-weeks on chow diet the increase of HDL-C in the female KO mice was not as prominent (+27%) as in the other data points, and there was hardly any PL elevation detectable (Fig. 3). The HDL-C and PL increase (females: TC, +51%; PL, +30%; males: TC, +40%; PL, +35%) was detectable in both genders at the starting point (data not shown). Despite the observed increase of HDL-C and –PL in the KO animals, it was not possible to detect a consistent particle size change in the size exclusion chromatography, due a limited resolution of the column set-up in this molecular weight region. The TG increase observed in the female Osbpl8KO mice on chow diet, and in male KO mice after the Western diet, localized almost exclusively to the very-low-density lipoprotein fractions (Fig. 3). The apolipoprotein composition of chow diet-fed WT and KO animal plasma was further investigated by Western blot analysis with specific antibodies against mouse apoA-II, apoC-III and apoE (Fig. 4A). No difference between the genders or the genotypes was observed in apoA-II levels. ApoC-III was more abundant in female than male mice, but no difference between genotypes was detected. Interestingly, an elevated level of apoE was present in the plasma of male Osbpl8KO mice as compared to WT males, while such increase was not detectable for the female gender (Fig. 4A). Analysis of apoE in the lipoprotein fractions of the mice localized the increase of apoE in male KO animals to the fractions where HDL particles elute (Fig. 4A, bottom panel), which also showed an elevated cholesterol and PL content (Fig. 3); apoE was undetectable in the VLDL fractions (data not shown). In Western diet-fed animals a similar increase of plasma and HDL apoE was evident in the male but not female KO mice, which rather showed a tendency of reduced HDL apoE content as compared to WT littermates (Fig. 4B).

### Hepatic lipid Content of the Osbpl8KO Animals

The liver tissue total TC, PL, and TG concentrations of the WT and Osbpl8KO mice were determined, revealing a significant elevation (from 5.5±0.4 to 7.1±0.3 µmol/g, p = 0.01) of TC in the tissue of Western diet-fed female (but not male) KO mice as compared WT littermates. Additionally, in both female and the male KO mice on chow diet a statistically insignificant tendency (p = 0.12) of elevated hepatic TG content was evident (Table 2).

**Table 2:** Liver tissue lipid content of the WT and Osbpl8KO mice.

	Triglycerides µmol/g(Mean±s.e.m.)	p-value*	Cholesterol µmol/g(Mean±s.e.m.)	p-value	Phospholipids µmol/g(Mean±s.e.m.)	p-value
Chow diet						
Female WT	7.1±0.5	0.12	4.2±0.1	0.96	0.91±0.06	0.45
Female KO	8.4±0.5		4.1±0.4		0.98±0.07	
Male WT	6.1±0.2	0.12	3.9±0.2	0.19	1.16±0.08	0.98
Male KO	9.0±1.6		4.3±0.2		1.16±0.04	
Western diet						
Female WT	20.0±2.6	0.32	5.5±0.4	0.01	1.16±0.07	0.94
Female KO	23.8±2.3		7.1±0.3		1.16±0.03	
Male WT	24.9±5.0	0.85	4.8±0.3	0.77	0.97±0.10	0.89
Male KO	23.5±4.3		4.9±0.2		0.95±0.02	

* T-test, comparison between genotypes, n = 6.

### Body Weight of the Animals

During the Western but not the chow diet, the weight of WT and Osbpl8KO mice of both genders increased significantly, with no difference observed between the genotypes (Table S2).

### Plasma LCAT, HL, LPL and PLTP Activity of the Osbpl8KO Mice

To search for putative explanations for the observed increase of HDL, we assayed the activities of plasma HDL modifying proteins, LCAT [14], PLTP [15], and HL [16]. In addition, LPL activity which can indirectly affect HDL [17], was determined. LCAT, HL, and LPL activity was measured from plasma of chow-diet fed animals, the lipases from plasma collected after intravenous heparin injection, while PLTP activity was determined for all plasma specimens of the diet experiment. No statistically significant differences in LCAT or HL activity were detected between Osbpl8KO and WT animals of either gender, whereas LPL activity was significantly reduced (by 38%) in the Osbpl8KO females as compared to WT animals (Table 3), consistent with the elevated plasma TG levels observed in these animals (Table 1). Interestingly, PLTP activity was reduced in both female (by 44%, p<0.05) and male (by 39% p = 0.05) KO animals on regular chow, but not on Western diet.

**Table 3:** Plasma PLTP and LCAT activity levels at t = 0 (A), PLTP activity level at t = 5 weeks (B), and HL/LPL activity in post-heparin plasma of chow-fed mice (C).

t = 0	PLTP activityµmol/ml/h(Mean±s.e.m.)	p-value	LCAT activityµmol/ml/h(Mean±s.e.m.)		p-value	
Female WT	18.00±1.70	0.60*	10.3±0.86		0.16	
Female KO	19.25±1.59		7.5±0.56			
Male WT	14.58±1.40	0.57	6.1±0.41		0.47	
Male KO	15.70±1.33		6.6±0.45			
**t = 5 weeks**	**PLTP activity** **µmol/ml/h** **(Mean±s.e.m.)**	**p-value**				
Western diet						
Female WT	17.45±2.38	1				
Female KO	17.44±1.49					
Male WT	24.94±3.02	0.68				
Male KO	23.42±1.84					
Chow diet						
Female WT	12.42±1.31	<0.05				
Female KO	6.92±2.06					
Male WT	12.92±1.50	0.05				
Male KO	7.87±1.69					
**Chow diet**	**HL activity** **µmol FFA/ml/h** **(Mean±s.e.m.)**	**p-value**	**LPL activity** **µmol FFA/ml/h (Mean±s.e.m.)**		**p-value**	
Female WT	8.68±0.79	0.11	26.00±0.99		<0.05	
Female KO	11.44±0.49		16.22±0.97			
Male WT	7.75±0.19	0.19	17.4±0		0.15	
Male KO	8.48±0.45		15.06±0.47			

* T-test, comparison between genotypes, n = 4–6 (A), n = 6 (B,C).

### HDL Catabolism in the Osbpl8KO Mice

**Figure 5:**
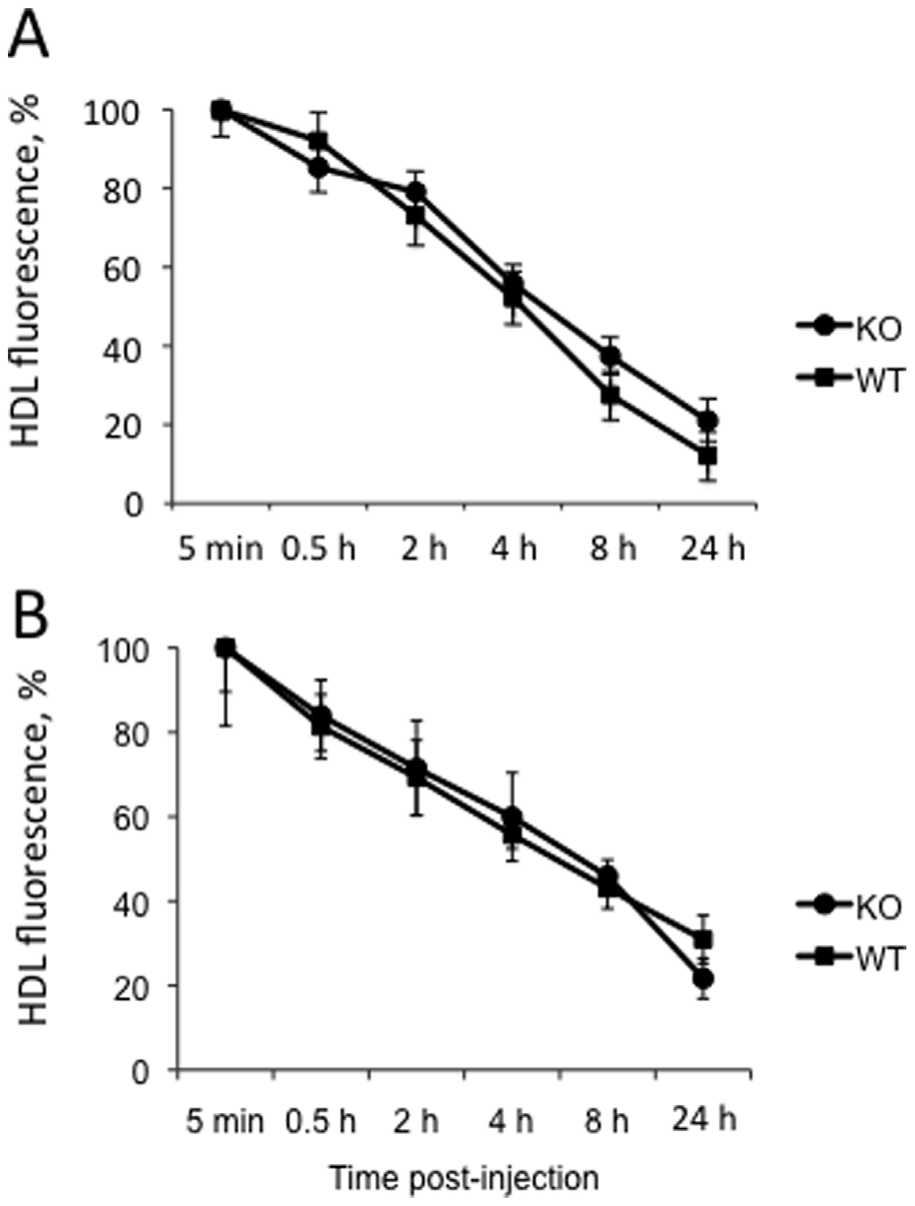
HDL catabolism in Osbpl8KO mice. Chow-fed WT and Osbpl8KO (n = 4–6) female (A) or male (B) animals were injected with Alexa568-labeled mouse HDL (40 µg protein/animal), and the fluorescence signal (y-axis; mean ± s.e.m., % of starting value at 5 min post-injection) in their plasma as a function of time (x-axis) was measured as specified in Materials and Methods.

To assess the possibility that the HDL elevation detected in the Osbpl8KO mice could be due to impaired catabolism of HDL particles, we injected groups of chow-fed WT and Osbpl8KO animals with Alexa568-labeled WT mouse HDL, and followed the decay of fluorescence in plasma during a time course for up to 24 h. The HDL fluorescence decay curves were practically identical, thus revealing no difference in the HDL fractional catabolic rate (Fig. 5).

### Cholesterol Biosynthetic and Absorption Markers in the Plasma of Ospbl8KO Mice

The previous findings that adenoviral ORP8 overexpression in mouse liver reduced plasma total cholesterol, and excess ORP8 in HuH7 hepatocytes dampened cholesterol biosynthesis [12], prompted us to analyze non-cholesterol sterol markers of cholesterol biosynthesis and absorption in the plasma of fasted chow-fed WT and Osbpl8KO animals. This analysis revealed a gender-specific, significant increase of the cholesterol biosynthetic markers cholestenol, desmosterol, and lathosterol in the male KO mice as compared to WT controls (Table 4), suggesting enhanced mevalonic acid pathway activity in these animals. No significant differences were observed between the WT and KO mice in the plasma levels of sterol absorption markers cholestanol, campesterol, sitosterol, or avenasterol (Table 4), consistent with the view that intestinal sterol absorption is not significantly affected by Osbpl8 deficiency.

**Table 4:** Non-cholesterol sterols in the plasma of WT and Osbpl8KO mice (n = 6).

µg/100 ml(Mean±s.e.m.)	Female WT	Female KO	p-value#	Male WT	Male KO	p-value
Cholestanol	399.30±8.81	337.61±14.37	0.978	504.22±22.25	472.62±23.28	0.469
Cholestenol	22.00±3.01	29.33±2.50	0.090	25.83±1.72	50.67±9.02	0.0221*
Desmosterol	82.50±7.98	84.20±12.20	0.613	130.67±9.88	296.17±42.17	0.0034**
Lathosterol	18.00±2.08	22.83±2.04	0.128	29.00±2.31	52.17±7.75	0.0168*
Campesterol	2066.50±274.82	2288.33±121.50	0.477	2990.00±190.85	3176.50±437.17	0.704
Sitosterol	699.33±100.63	714.67±42.50	0.891	991.50±57.42	1189.00±129.72	0.194
Avenasterol	66.17±6.05	73.50±5.33	0.384	85.00±4.97	105.50±10.61	0.111
Squalene	16.67±3.09	18.83±2.30	0.524	25.33±3.29	25.60±2.78	0.502

# T-test, comparison between genotypes; *p<0.05, **p<0.01.

### Hepatic Expression of mRNAs and Proteins Involved in Lipid Metabolism

**Figure 6:**
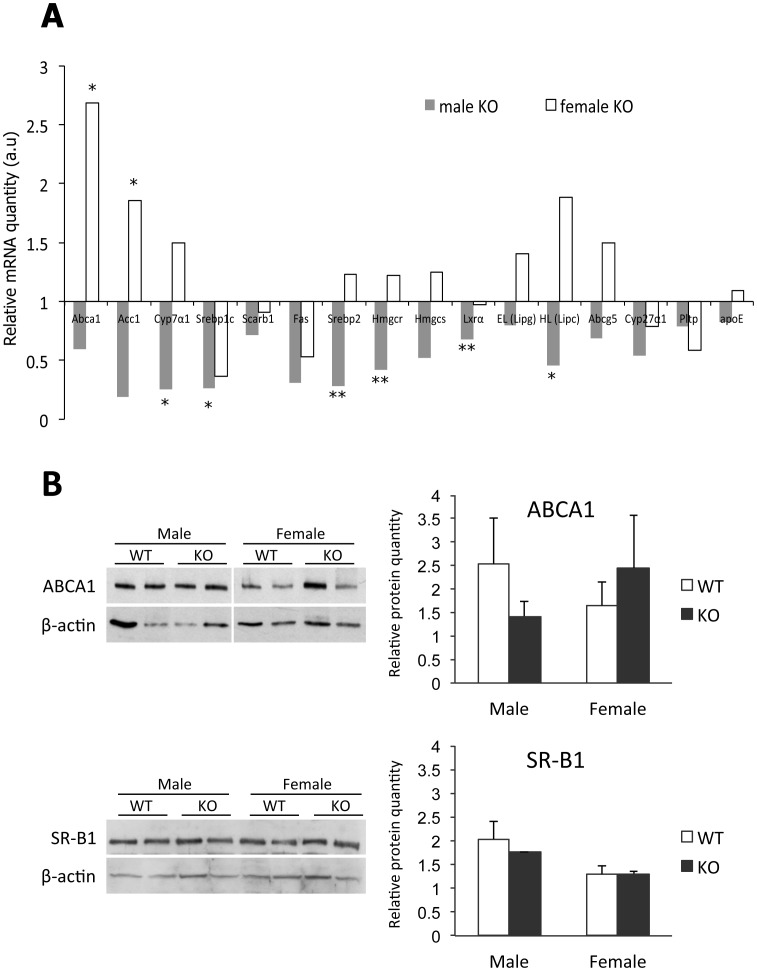
Liver mRNA and protein expression analysis of chow-fed Osbpl8KO mice. A: qPCR analysis of the quantity of the mRNAs identified at the bottom in chow-fed KO females (open bars) and males (closed bars). The mRNAs were quantified using ribosomal protein 36B4 message as a housekeeping reference. The data are expressed relative to quantity in littermate WT animals of the same gender, and represent mean ± s.e.m. (n = 6; *p<0.05, **p<0.01, T-test). B: Western blot analysis of ABCA1 and SR-B1 proteins in WT and KO mouse liver. The blots were probed with anti-β-actin as a loading control. Densitometric quantification of the Western blot data is shown on the right. The results were normalized against β-actin. The data represents mean ± s.e.m. (n = 4).

The fact that an HDL elevation in Osbpl8KO mice was detected in both chow and Western diet fed animals suggested that the effect is not due to a difference in lipid absorption, but rather in hepatic HDL synthesis or in HDL catabolism. However, we found no defect in the capacity of the KO animals to catabolize HDL (Fig. 5). We therefore analyzed by qPCR a number of mRNAs encoding proteins which could potentially impact hepatic HDL synthesis, as well as the hepatic protein levels of ABCA1, a key player in HDL biosynthesis and secretion [18], [19], and SR-B1, which acts as a receptor in the selective uptake of HDL cholesteryl esters by the liver and steroidogenic tissues [20]. The mRNA quantification revealed only modest alterations in the Osbpl8KO mice, which in many cases reached statistical significance in only one gender, the other gender often showing a change or a tendency in the opposite direction (Fig. 6A). As an example, the Abca1 mRNA was significantly up-regulated in the liver of female KO mice, while an opposite but non-significant tendency was evident in males. Similarly, the mRNA for acetyl-coenzyme A carboxylase 1 (Acc1) was significantly up-regulated in female KO mice, but showed a tendency of down-regulation in KO males, whereas the lipogenic transcription factor regulating this gene, sterol regulatory element binding protein 1c (Srebp-1c), was down-regulated in male KOs and showed a tendency of reduction also in females. Of the mRNAs encoding lipases that control HDL levels, we observed a significant down-regulation in hepatic lipase (Lipc) mRNA in male animals, and an opposite tendency in females. Other significant mRNA changes observed were a down-regulation of liver X receptor-α (Lxrα) and its target gene cholesterol 7α-hydroxylase (Cyp7a1), as well as Srebp-2 and its target 3-hydroxy-3-methylglutaryl-coenzyme A reductase (Hmgcr) in male KO animals, the latter observation being notably inconsistent with the observed increase of cholesterol biosynthesis markers in the plasma of the KO males. In accordance with the mRNA findings, there were no differences between the genotypes in hepatic SR-B1 (Scarb1) protein levels as determined by Western analysis. The ABCA1 protein quantity was moderately elevated in KO females and reduced in KO males as compared to WT littermates; These differences, however, did not reach statistical significance (Fig. 6B). The hepatic mRNA and protein findings thus did not provide any clearly defined mechanistic explanations for the observed lipid phenotypes. However, they further support the conclusion that Osbpl8 deficiency has pleiotropic and gender-specific effects on lipid homeostatic machineries and suggest a multi-faceted regulatory role of the ORP8 protein.

### Immunohistochemistry (IHC) Analysis of ABCA1, apoA-I and apoE in Osbpl8KO Mouse Liver and Kidney

To assess possible changes in the hepatocellular distribution of ABCA1, a major controller of HDL biogenesis, as well as the apolipoproteins apoA-I and apoE, we carried out IHC of the liver of chow diet-fed WT and Osbpl8KO mice. The morphology and the ABCA1 staining patterns of the WT and KO tissues were indistinguishable: A relatively even ABCA1 immunoreactivity in the hepatocytes was observed, with some cells showing a more intense staining than others (Fig. S1), similar to the pattern previously reported [21]. ApoA-I staining was observed within the sinusoidal compartments, apparently reflecting plasma immunoreactivity (Fig. S2). Also the apoE antibody stained the sinusoids, but displayed an additional intracellular staining of granular-like structures in hepatocytes, possibly representing secretory organelles, with no detectable difference between the WT and KO tissues (Fig. S3). Moreover, we stained apoA-I and apoE in WT and Osbpl8KO kidney, an organ with an important role in HDL catabolism. Staining with both antibodies was localized in the vascular lumen compartments and the glomeruli, with no detectable difference between the genotypes (data not shown).

### Nascent HDL Produced by ORP8-deficient Hepatocytes

**Figure 7:**
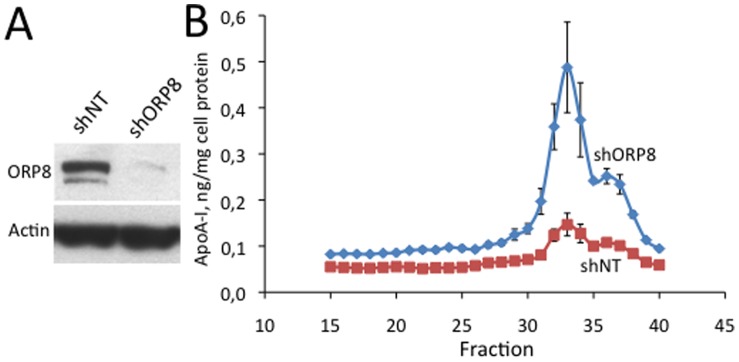
Nascent HDL produced by ORP8-deficient hepatocytes. Nascent HDL particles secreted into serum-free culture medium by Hepa1-6 cells with ORP8 stably silenced with shRNAs (shORP8) or expressing non-targeting shRNA (shNT) were fractioned on a Superose 6 HR 10/30 column. ApoA-I in the fractions was quantified with ELISA, and the associated phospholipids were analyzed by ESI-MS. A. Western blot of the Hepa1-6 cell pools probed with anti-ORP8 (ORP8) and anti β-actin (Actin), showing the efficiency of ORP8 knock-down. B. ApoA-I in the Superose 6 HR 10/30 fractions. The data is normalized per mg total cell protein in the cultures. The data represents mean ± s.e.m. (n = 3).

To assess a putative role of ORP8 in the production and lipidation of nascent HDL produced by hepatocytes, we employed the mouse hepatoma cell line Hepa1-6, which was subjected to shRNA-mediated silencing of Osbpl8 (Fig. 7A). The nascent HDL secreted into the culture medium was fractioned by size exclusion chromatography and its phospholipid (PL) molecular species profile was analyzed by ESI-MS. The results revealed a marked increase of small apoA-I containing particles secreted into the growth medium by ORP8 knock-down (shORP8) cells as compared to controls expressing non-targeting shRNA (shNT)(Fig. 7B). The particles appeared in the size exclusion chromatography as a double peak with the apparent molecular masses of 75 and 25 kDa. In ESI-MS analysis of phospholipids in the pooled apoA-I containing fractions (nos 30–39), 13 phosphatidylcholine (PC) species, of which PC34∶1, PC36∶2, PC36∶1, and PC32∶1 were most abundant, as well as sphingomyelin (SM) 16∶0 were detectable. The analysis revealed no difference in the phospholipid molecular species composition between the apoA-I-containing fractions from shNT and shORP8 cells (data not shown). These results suggest that, even though the shORP8 hepatocytes appear to display an increased capacity to secrete nascent HDL, the phospholipids associated with apoA-I secreted by ORP8-depleted and control hepatocytes do not differ significantly.

## Discussion

In this study we created the first viable Osbpl gene knock-out mouse model and characterized the effects of Osbpl8 deficiency on mouse lipoprotein metabolism. In the KO mice, no immunoreactive ORP8 protein was present in tissues/cells earlier reported to express ORP8 at highest levels: liver, brain, kidney, spleen, and peritoneal macrophages [10], suggesting that the knock-out generated by using gene-trapped ES cells is global, and that Osbpl8 is not essential for viability.

Analysis of the Osbpl8KO mouse plasma lipids and lipoproteins revealed a significant increase of cholesterol and choline phospholipids as compared to wild-type littermates, reflecting their increase specifically in HDL particles. The HDL increment was prominent in both genders on Western diet, while in chow diet-fed animals it was less pronounced in females than males. The HDL increase was accompanied by an elevated level of apoE in the plasma and HDL of male KO mice. Adenoviral overexpression of Osbpl8 in mouse liver was previously shown to result in a reduction of plasma and liver tissue cholesterol and TG [12], consistent with the present increase of plasma cholesterol upon Osbpl8 deficiency. However, analysis of plasma lipoprotein fractions was not reported in the overexpression study, which makes a detailed comparison of the results impossible. We found no evidence for altered LCAT or HL activity between the WT and Osbpl8 animals, which might have explained the observed HDL elevation [14], [16]. Interestingly, the KO mice displayed a reduced plasma PLTP activity, but only on chow diet. PLTP deficiency was previously shown to reduce, not increase, the plasma HDL [22]. This, together with the diet-dependency of the effect on PLTP, makes it unlikely that altered PLTP activity could be the cause of the HDL elevation observed in both chow and Western diet-fed animals. Analysis of the fractional catabolic rate of exogenously administered mouse HDL revealed no difference between the genotypes, suggesting that the Osbpl8 deficiency does not impair the capacity of the animals to catabolize HDL. The HDL elevation was evident in the Osbpl8 KO mice independently of the diet (albeit less prominent in female KO mice on chow diet), suggesting that it is not due to a difference in intestinal lipid absorption. The fact that HDL lipids were in the present study elevated more strongly than apoA-I suggests that the lipidation of HDL particles is enhanced in the KO animals. Approximately 70–80% of HDL in mouse is of hepatic origin [23], [24], and ABCA1 plays a key role in the lipidation and secretion of nascent apoA-I in hepatocytes [18], [19]. However, analysis of hepatic ABCA1 mRNA and protein levels, or immunohistochemical analysis of ABCA1 distribution, provided no direct synthesis-related explanation to the HDL elevation detected in both genders. Furthermore, no significant alteration in hepatic apoE mRNA expression was detected, which could have explained the observed increase of apoE in the plasma and HDL of male KO mice. Interestingly, the male KO mice displayed elevated plasma levels of non-cholesterol sterol markers for sterol biosynthesis, suggesting a gender-specific enhancement of hepatic mevalonic acid pathway activity. This finding appears to be in line with the previous report where ORP8 overexpression in mouse liver was shown to dampen the expression of cholesterol biosynthetic pathway genes controlled by SREBP-2 [12]. However, we found no evidence for a corresponding increase of HMGCoA reductase or synthase mRNAs in the male KO mice, suggesting that the putative enhancement of cholesterol biosynthesis *in vivo* may occur via a different, possibly post-transcriptional mechanism.

Thus far the mechanisms of ORP function have remained poorly understood. However, a number of functional clues point to action as lipid sensors at contacts where the endoplasmic reticulum (ER) communicates with other membraneous organelles, termed membrane contact sites, MCS [25]–[28]. Another emerging theme is the interaction of ORPs with phosphoinositides, particularly phosphatidylinositol-4-phosphate (PI4P), which mediates the membrane association of ORPs, a process regulated by the binding of sterol within the OSBP-related ligand-binding (ORD) domain of the proteins [29]–[31]. ORP8 has a C-terminal trans-membrane segment that targets the ER and the nuclear envelope [10], while its N-terminal pleckstrin homology domain region has the capacity to target the plasma membrane (T.Vihervaara and V.M.Olkkonen, unpublished). We therefore envision that part of ORP8 may act at ER-plasma membrane contacts, where it could, guided by sterol and phosphoinositide signals, regulate lipid metabolizing or remodeling enzymes [27], lateral lipid domains [32], or signaling events [13]. Interestingly, we found in ORP8-depleted mouse hepatocytes a significant increase of nascent HDL secreted into the culture medium, which could reflect modulation of signaling events that control HDL biogenesis. It has also been suggested that ORP8 may, via interaction with nucleoporin Nup62, modify nuclear functions involving the transcriptional control of lipid metabolism [12]. However, the present mRNA analyses only revealed mild and gender-specific effects of Osbpl8 deficiency on genes of lipid metabolism, with no apparent correlations with the observed lipid phenotypes, suggesting that the impacts of the knock-out rather reflect altered post-transcriptional regulation. Yang et al. [33] reported than many hepatic genes in mouse show sexual dimorphism. Furthermore, the largest changes in gene expression between females and males were observed in genes involved in steroid and lipid metabolism. The gender-specific impacts of Osbpl8 deficiency on LPL activity, cholesterol biosynthesis markers, and on hepatic gene expression most likely reflect such sexual dimorphism, and underscore the crucial importance of investigating the two genders separately.

The present study represents the first report of a viable Osbpl knock-out mouse model. We demonstrate a function of endogenous ORP8 as a controller of mouse plasma HDL. In addition to the impact on HDL, the results reveal pleiotropic, gender- and/or diet-specific effects of Osbpl8 deficiency on several parameters of lipid/lipoprotein metabolism. Understanding in detail the molecular mechanisms through which ORP8 exerts its multiple effects on lipid homeostatic regulation is a challenging aim of future investigations, for which the present KO mouse model will be an instrumental tool.

## Methods

### Generation of Osbpl8 Knock-out (KO) Mice

An E14 mouse embryonic stem (ES) cell line containing a gene trap construct in the Osbpl8 gene (clone DD00748) was obtained from the Sanger Institute Gene Trap Resource (SIGTR, [34]). The ES cells were microinjected in blastocyst stage C57Bl/6 embryos producing germline chimeric mice. The chimeric mice were bred with C57Bl/6 mice to obtain heterozygous animals and again homozygous mice in the F2 generation. For this study the heterozygous animals were repeatedly back-crossed with C57Bl/6 to bring the trapped Osbpl8 gene in C57Bl/6 background. To speed up the process, the fathers of each generation were selected based on data from microarray SNP genotyping carried out using Illumina (San Diego, CA) arrays (GT-104-1213), to identify males with the largest proportion of C57Bl/6 strain chromatin. After 6 generations of breeding the strain used for the present experiments was >98% C57Bl/6 congenic. The exact gene trap (GT) insertion point was determined by performing genomic PCR using GT and Osbpl8 specific primers. The GT vector, which consists of a splice acceptor site linked to a β-geo selectable marker, was found to be inserted into the 5^th^ intron of the Osbpl8 gene on chromosome 10. With 3 specific primers, we distinguished wild-type (WT), heterozygote (+/−) and homozygote (KO) mice using genomic PCR. The mouse work was conducted under licenses (STH715A and STH847A) granted by the National Animal Experiment Board (ELLA) under Regional State Administrative Agency for Southern Finland, in conformity with the Public Health Service (PHS) Policy on Humane Care and Use of Laboratory Animals. The ELLA is the appropriate authority to authorize animal experimentation; Approval by the institution is not necessary.

### Mouse Genotyping

Genotyping animals from gDNA was performed by PCR, using a set of 3 primers targeting (i) an intronic area before the GT insertion, (ii) the GT insert, and (iii) intronic area after the GT insertion: Fwd1, 5′-TAgTggTTTAgTCTCTgCgAg-3′, Rev1 5′-AgAAgCAggCCACCCAACTgA-3′, and Rev2, 5′-TAACgAgCAAACATgAgCCA-3′. A 4-fold excess of Fwd1 primer relative to the other two was used. The PCR product sizes are 162 bp for the pair Fwd1-Rev1 and 341 bp for Fwd1-Rev2.

### Mouse Maintenance and Diet

Mice were maintained at Helsinki University Laboratory Animal center premises at a temperature of 21–22°C, 52% humidity and light cycle of 12∶12 h, on Global 16% rodent diet 2916 (Teklad, Harlan Laboratories, Inc., Indianapolis). For a diet experiment, groups of Osbpl8 KO and WT littermate female and male animals (n = 6 for each group, 4 groups per diet) at the age of 13 weeks were bled from *vena saphena* after 4 h fast and either continued for 5 weeks on chow diet (a total of 24 animals), or were fed ad libitum a high-fat (Western) diet (a total of 24 animals) containing: 17.3% protein, 48.5% carbohydrate, 21.2% fat, 0.2% cholesterol, contents by weight (Harlan adjusted calories diet, TD.88137). At the end, the mice were again fasted for 4 h and terminated with CO_2_, followed by withdrawal of blood through heart puncture and snap freezing of tissue specimens in liquid N_2_. The blood was drawn in EDTA tubes and plasma was separated by centrifugation.

### Plasma Lipid Analyses

Plasma triglycerides (GPO-PAP 1488872 kit, Roche Diagnostics, Mannheim, Germany), total cholesterol (CHOD-PAP 1489232 kit, Roche Diagnostics) and choline-containing phospholipids (Wako Chemicals, Richmond, VA, Phospholipids B-kit or Daiichi Pure Chemicals, Tokyo, Japan, Pureauto S PL-kit) were measured using enzymatic methods.

### Size-exclusion Chromatography of Plasma Lipoproteins

Plasma lipoproteins were fractionated by fast-performance liquid chromatography (FPLC; Merck-HPLC System) using Superose 6 HR 10/30 size-exclusion chromatography column (GE Healthcare, Buckinghamshire, UK). The column was equilibrated with 10 mM Na-phosphate buffer, pH 7.4 containing 140 mM NaCl, and then 110–150 µl of plasma was applied to the column with a flow rate of 0.5 ml/min, fractions of 0.5 ml were collected, and analyzed for cholesterol, triglycerides, phospholipids and apoA-I. For each group of 6 animals, two plasma pools of 3 animals each were analyzed.

### Assay of Plasma PLTP Activity

For the radiometric PLTP activity assay, phosphatidylcholine (PC) liposomes were prepared essentially as described by Damen et al. [35] and the activity assay was carried out as described by Jauhiainen and Ehnholm [36]. Prior to analysis, the fasting plasma samples were diluted 1∶10 with assay buffer and 4 µl of the dilution was used for phospholipid transfer assay.

### ApoA-I Quantification

Mouse apolipoprotein A-I (apoA-I) was quantified by a sandwich enzyme-linked immunosorbent assay (ELISA) [37].

### Assays for Lecithin:cholesterol Acyltransferase (LCAT), Lipoprotein Lipase (LPL) and Hepatic Lipase (HL) Activity

LCAT activity in plasma of fasted (4 h) animals was assessed by measuring cholesterol esterification activity in individual plasma samples using exogenous proteoliposome [^3^H]cholesterol-HDL discs as the substrate [38]. Post-heparin plasma was collected after tail vein injection of 100 IU heparin per kg body weight, and plasma LPL and HL activities were measured as previously described [39], [40]. Briefly, [Carboxyl-^14^C]-Triolein (S.A. 2.2 GBq/mmol, PerkinElmer, Waltham, MA) and glyceryl trioleate (Sigma-Aldrich, St. Louis, MO) emulsified in the presence of gum arabic was used as substrate. Post-heparin plasma samples (15–25 uL) were incubated with the substrate and human serum (as source for apoC-II, LPL cofactor) for 1 hour at 37°C. Hepatic lipase was analyzed in the presence of 1 M NaCl to inhibit LPL activity. Radioactivity was measured by liquid scintillation counting (Wallac LS Counter, Turku, Finland). LPL and HL activities are expressed as µmol FFA/mL/h.

### Non-cholesterol Sterol Analysis

Fasting plasma cholesterol, cholesterol precursors (squalene, cholestenol, desmosterol, and lathosterol), campesterol, sitosterol and avenasterol (plant sterols), and cholestanol, a metabolite of cholesterol, were quantified from nonsaponifiable plasma material by capillary gas-liquid chromatography (GLC) (Agilent 6890N Network GC System, Agilent Technologies, Wilmington, DE) equipped with a 50 m long nonpolar Ultra 2 capillary column (5% Phenyl-methyl siloxane) with 5α-cholestane as internal standard [41]. The coefficients of variation are as follows: cholesterol 3.2%, cholestanol 2.7%, desmosterol 6.0%, lathosterol 3.7%, campesterol 1.8%, and sitosterol 2.4%, respectively. The plasma values are expressed as concentrations (µg/dl). The concentrations of cholesterol precursors reflect whole-body cholesterol synthesis, and those of plant sterols and cholestanol reflect cholesterol absorption [42], [43].

### Liver Tissue Lipid Analyses

Sections of mouse liver tissue were excised, snap-frozen in liquid nitrogen and stored at −70°C. Cellular lipids were extracted from the tissue by the method of Folch et al. [44], and triglycerides were measured as glycerol after chloroform–methanol extraction and hydrolysis. Briefly, liver tissue (approximately 50–100 mg) was homogenized and sonicated in 1 ml 95% methanol and mixed with 2 ml chloroform. The organic phase was washed with 0.9% NaCl solution and dried under nitrogen. The residuals were dissolved in 200 µl of tetraethylammoniumhydroxide (diluted 1∶28 with 95% ethanol) and incubated at 60°C for 30 min with 200 µl of 0.05 M HCl. The formed glycerol was measured enzymatically using a commercial triglyceride analysis kit (GPO-PAP 1488872, Roche Diagnostics). Total cholesterol and choline-containing phospholipids (PC, lyso-PC, SM) were measured for the same tissue specimens from the solvent phase after initial chloroform–methanol extraction, using the enzymatic assays specified above for plasma lipids.

### Western Blotting

Tissues or peritoneal macrophages were homogenized in 50 mM Tris-HCl, pH 7.4, 150 mM NaCl, 1mM EDTA, 1% NP-40, 0.25% Na-deoxycholate, 1% SDS, protease inhibitor cocktail (Roche Diagnostics). After protein determination with the Pierce (Dallas, TX) BCA assay, protein specimens were boiled for 5 min in reducing Laemmli loading buffer. Similarly, HDL peak fractions from plasma size-exclusion chromatography on Superose 6 HR 10/30 were boiled in loading buffer. The specimens were loaded on 10% homogeneous or 4–15% gradient SDS-polyacrylamide gels (BioRad, Hercules, CA). The separated proteins were electrotransferred onto Hybond-C nitrocellulose membranes (GE Healthcare/Amersham, Buckinghamshire, UK). Unspecific binding of antibodies was blocked with 5% (w/v) fat-free milk in 10 mM Tris-HCl, pH 7.4, 150 mM NaCl, 0.1% Tween-20. Primary antibodies diluted in the same buffer were incubated overnight at +4°C, and the bound antibodies were detected using peroxidase conjugated secondary IgGs (Jackson Immunoresearch, West Grove, PA), followed by visualization with SuperSignal West Pico chemiluminescence substrate (Pierce) and densitometric quantification normalized using the β-actin signal. The rabbit ORP8 antibodies used were described by Yan et al. [10]. The ABCA1 (NB400-105) and SR-B1 (NB400-101) antibodies were from Novus Biologicals (Littleton, CO) and anti-β-actin (A2066) from Sigma-Aldrich. The rabbit apoE antibody was described in [45]. The apoA-II antibodies (K23400R) were from Biodesign International (Memphis, TN) and apoC-III antibodies from Isis Pharmaceuticals (Carlsbad, CA).

### Immunohistochemistry

Immunohistochemical stainings of tissue paraffin sections were performed using the rabbit ABCA1 and apoE antibodies specified above, and rabbit anti-apoA-I (K23001R) from Meridian Life Science (Memphis, TN). The primary antibodies were detected using the avidin–biotin complex system (Vectastain ABC Elite rabbit kit, Vector Laboratories, Burlingame, CA). Brown 3,3′-diamino-benzidine (DAB, Sigma-Aldrich), was used as chromogen. Sections were counterstained with Mayer's hematoxylin. The stained samples were photographed with a digital camera (DFC480, Leica, Wetzlar, Germany) attached to a Leica DM4500B microscope. Specificity of the signals observed was controlled for by carrying out similar stainings in the absence of primary antibody, (data not shown).

### Quantitative Real-time RT-PCR (qPCR)

Tissue total RNA was isolated by using the Pure Link RNA Mini® kit (Ambion, Austin, TX) and reverse transcribed with Superscript III® (Invitrogen, Carlsbad, CA). qPCR was carried out on a Roche Lightcycler 480 II instrument by using the SYBR Green master mix from Roche, with primers specified in Table S1. The ribosomal protein 36B4 mRNA was used as a reference housekeeping message. Relative mRNA quantities were calculated by using the ΔΔC_T_ method.

### HDL Catabolism

HDL (1.0 mg of protein) purified from C57Bl/6 mouse plasma by ultracentrifugation in KBr, density 1.063–1.21 g/ml [46] was labeled with the Alexa Fluor®568 Protein Labeling Kit (A10238; Molecular Probes, Eugene, OR), followed by purification of the labeled particles on a Superose 6 HR 10/30 column (GE Healthcare). The HDL (40 µg protein/animal) was injected into the tail vein of chow-fed WT or ORP8 KO mice, followed by blood sampling into EDTA tubes at 5 min, 30 min, 2 h, 4 h, and 8 h post-injection. After 24 h the mice were terminated with CO_2_ and blood was collected through heart puncture. Plasma was separated as above, and fluorescence in specimens diluted 1/20 with PBS was determined by using the PHERAstar fluorometer (BMG Labtech, Germany). After the tail vein injection (5 min, 4 h, and 4 h time points), we controlled the distribution of the Alexa label in plasma by sixe exclusion chromatography as described above. This analysis revealed that at all three time points 70–80% of the fluorescence localized in the HDL fractions (data not shown).

### Osbpl8 Silencing in Hepa1-6 Cells and Nascent HDL Production

Osbpl8 was stably silenced in the mouse hepatoma cell line Hepa1-6 (CRL-1830, American Type Culture Collection, Manassas, VA) by using an Osbpl8-specific Sigma-Aldrich shRNA lentivirus TRCN 0000105248, as described in [13]. Non-Target shRNA expressing lentivirus (SHC002V) was used as a control.

### Nascent HDL Enrichment and ESI-MS Lipid Analysis

The Hepa1-6 cell pools cultured on 10 cm dishes in Dulbecco’s Modified Eagle’s Medium (DMEM, Sigma-Aldrich), 10% foetal bovine serum, penicillin/streptomycin, were washed twice with PBS, and the medium was replaced with 10 ml of serum-free medium/dish. The medium (20 ml/specimen) was collected after 24 h and, after centrifugation (10 min, 500×g), concentrated with Microsep™ centrifugal device using 10K molecular weight cut-off ultrafiltration membrane according to the manufacturer’s instructions (Pall Life Sciences, Ann Arbor, MI), and fractioned on a Superose 6 HR 10/30 column (GE Healthcare) as specified above. Lipids in the fractions containing apoA-I (identified by Western analysis) were extracted by using the Folch protocol [44] and analyzed by electrospray ionization mass spectrometry (ESI-MS) as described in [47].

## Supporting Information

Figure S1 **Immunohistochemical staining of ABCA1 in representative sections of Osbpl8KO and wild-type (WT) mouse liver.**
(TIF)

Figure S2 **Immunohistochemical staining of apoA-I in representative sections of Osbpl8KO and wild-type (WT) mouse liver.**
(TIF)

Figure S3 **Immunohistochemical staining of apoE in representative sections of Osbpl8 and wild-type (WT) mouse liver.**
(TIF)

Table S1 **Oligonucleotide primers used for mRNA quantification by quantitative real-time reverse transcription-PCR.**
(DOCX)

Table S2 **Body weight of the WT and Osbpl8KO mice before (n = 12) and after (n = 6) the diets.**
(DOCX)
