# Supplementary figures and images for: Osbpl8 Deficiency in Mouse Causes an Elevation of High-Density Lipoproteins and Gender-Specific Alterations of Lipid Metabolism
Source: PLoS One. 2013 Mar 15;8(3):e58856. doi: 10.1371/journal.pone.0058856 (PMC3598917; doi:10.1371/journal.pone.0058856)

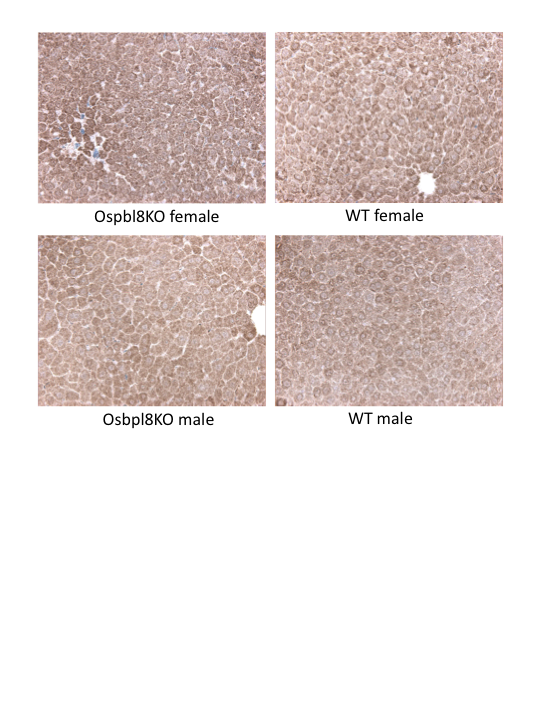

Supplement: Figure S1 — Immunohistochemical staining of ABCA1 in representative sections of Osbpl8KO and wild-type (WT) mouse liver. (TIF) [file pone.0058856.s001.tif]

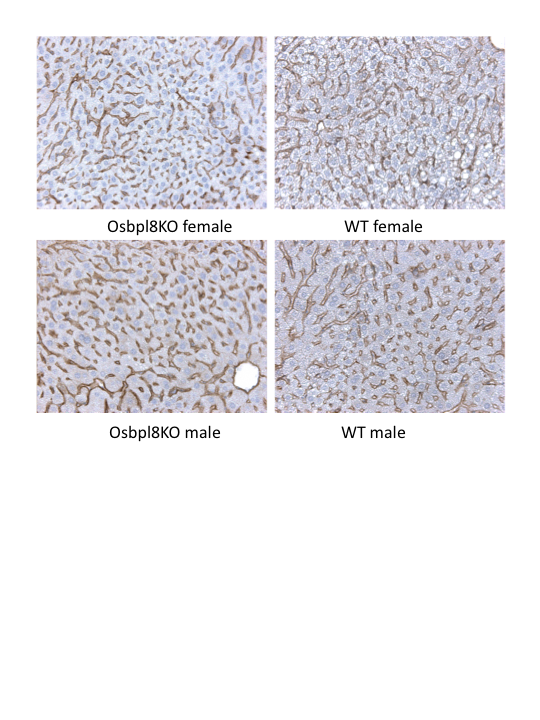

Supplement: Figure S2 — Immunohistochemical staining of apoA-I in representative sections of Osbpl8KO and wild-type (WT) mouse liver. (TIF) [file pone.0058856.s002.tif]

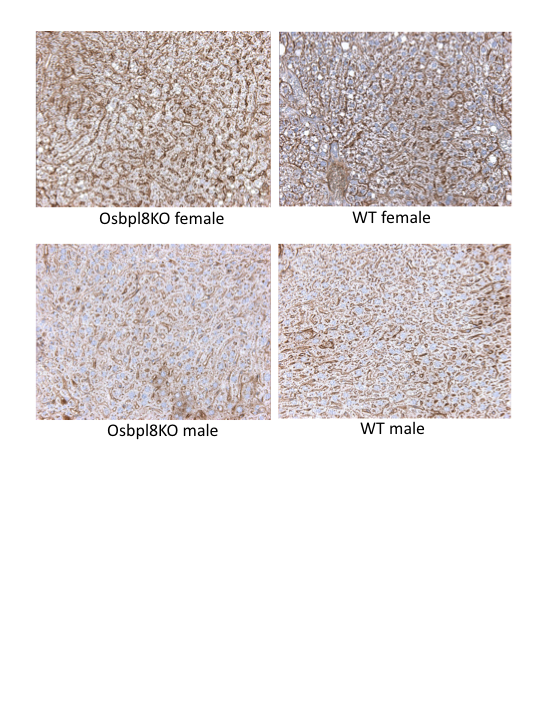

Supplement: Figure S3 — Immunohistochemical staining of apoE in representative sections of Osbpl8 and wild-type (WT) mouse liver. (TIF) [file pone.0058856.s003.tif]
