# Supplementary material for: Osbpl8 Deficiency in Mouse Causes an Elevation of High-Density Lipoproteins and Gender-Specific Alterations of Lipid Metabolism
Source: PLoS One. 2013 Mar 15;8(3):e58856. doi: 10.1371/journal.pone.0058856 (PMC3598917; doi:10.1371/journal.pone.0058856)
Supplement: Table S1 — Oligonucleotide primers used for mRNA quantification by quantitative real-time reverse transcription-PCR. (DOCX) [file pone.0058856.s004.docx]

**TABLE S1 Oligonucleotide primers used for mRNA quantification by quantitative real-time reverse transcription-PCR**

| mRNA | Forward primer 5'-3' | Reverse primer 5'-3' |
| --- | --- | --- |
| Abca1 | CgT TTC Cgg gAA gTg TCC TA | gCT AgA gAT gAC AAg gAg gAT |
| Acc1 | Tgg CTT gCA CCT AgT AAA ACA A | CCA CCg ACg gAT AgA TCg C |
| Cyp7α1 | AgC AAC TAA ACA ACC TgC CAg | gTC Cgg ATA TTC AAg gAT gCA |
| Srebp1c | AgC TgT Cgg ggT AgC gTC Tg | gAg AgT Tgg CAC CTg ggC Tg |
| Scarb1 | ggC TgC TgT TTg CTg Cg | gCT gCT TgA TgA ggg Agg g |
| Fas | gCT gCg gAA ACT TCA ggA AAT | AgA gAC gTg TCA CTC CTg gAC TT |
| Srebp2 | gCg TTC Tgg AgA CCA Tg gA | ACA Aag TTg CTC TgA AAA CAA |
| Hmgcr | CAC CTC TCC gTg ggT TAA AA | gAA gAA gTA ggC CCC CAA TC |
| Hmgcs | TTC CAA gCC CTg CTA AgA AA | TCC CAA gAC ATC CAT TCC TC |
| Lxrα | TCA gCA TCT TCT CTg CAg ACC gg | TCA TTA gCA TCC gTg ggA ACA |
| EL (Lipg) | CTg gAT ACg CTg gCA ACT TT | CAg CCT TCT gTT gAT gTC CA |
| HL (Lipc) | gAA ATC CCC TCC AAA TCT CC | gTA gCT CCA Agg CTT CTC CC |
| Abcg5 | Tgg ATC CAA CAC CTC TAT gCT | ggC Agg TTT TCT CgA TgA ACT |
| Cyp27α1 | CTA TgT gCT gCA CTT gCC C | ggg CAC TAg CCA gAT TCA CA |
| Pltp | ggA Agg CCg TCT CAg TgC TA | CgC ACg Aag TTg ATA CCC TCA |
| ApoE | Tgg Agg ACA CTA TGA Cgg AAG | gTT gCg TAg ATC CTC CAT gTC |
| 36B4 | ggA CCC gAg Aag ACC TCC TT | ggA CAT CAC TCA gAA TTT CAA |
