# Supplementary material for: Osbpl8 Deficiency in Mouse Causes an Elevation of High-Density Lipoproteins and Gender-Specific Alterations of Lipid Metabolism
Source: PLoS One. 2013 Mar 15;8(3):e58856. doi: 10.1371/journal.pone.0058856 (PMC3598917; doi:10.1371/journal.pone.0058856)
Supplement: Table S2 — Body weight of the WT and Osbpl8KO mice before (n = 12) and after (n = 6) the diets. (DOCX) [file pone.0058856.s005.docx]

**TABLE S2 Body weight of the WT and Osbpl8KO mice before (n=12) and after (n=6) the diets**

|  | Weight before diet/g  (Mean±s.e.m.) | p-value* | Weight after Chow diet/g  (Mean±s.e.m.) | p-value | Weight after Western diet/g (Mean±s.e.m.) | p-value |
| --- | --- | --- | --- | --- | --- | --- |
| Female WT | 21.73±0.49 | 0.53 | 24.2±1.05 | 0.087 | 25.07±1.10 | 0.26 |
| Female KO | 21.25±0.57 |  | 21.73±0.74 |  | 27.53±1.12 |  |
| Male WT | 29.63±0.27 | 0.69 | 31.5±0.61 | 0.61 | 36.85±1.34 | 0.56 |
| Male KO | 29.37±0.58 |  | 31.08±0.5 |  | 35.47±1.87 |  |

*T-test, comparison between genotypes
